# Melanocyte Colonization and Pigmentation of Breast Carcinoma: Description of Two Pathological Cases and Review of Literature

**DOI:** 10.3390/diagnostics11040709

**Published:** 2021-04-15

**Authors:** Angela Santoro, Giuseppe Angelico, Vincenzo Fiorentino, Qianqian Zhang, Saveria Spadola, Angela Carlino, Alejandro Martin Sanchez, Gianluca Franceschini, Gian Franco Zannoni, Antonino Mulè

**Affiliations:** 1 Unità di Ginecopatologia e Patologia Mammaria, Dipartimento Scienze della Salute della Donna, del Bambino e di Sanità Pubblica, Fondazione Policlinico Universitario A. Gemelli IRCCS, Largo A. Gemelli 8, 00168 Roma, Italy; angela.santoro@policlinicogemelli.it (A.S.); giuangel86@hotmail.it (G.A.); vincenzof.89@hotmail.it (V.F.); zhangqianqian0921@gmail.com (Q.Z.); saveriaspadola@hotmail.it (S.S.); angela.carlino@policlinicogemelli.it (A.C.); antonino.mule@policlinicogemelli.it (A.M.); 2 Department of Woman and Child Health and Public Health, Division of Breast Surgery, Fondazione Policlinico Universitario Agostino Gemelli IRCCS, Università Cattolica del Sacro Cuore, Largo Agostino Gemelli, 8, 00168 Rome, Italy; martin.sanchez@policlinicogemelli.it (A.M.S.); franceschinigianluca@gmail.com (G.F.); 3 Istituto di Anatomia Patologica, Università Cattolica del Sacro Cuore, Largo A. Gemelli 8, 00168 Roma, Italy

**Keywords:** breast carcinoma, melanocyte colonization, pigmentation, breast melanosis, melanoma

## Abstract

Colonization of breast carcinoma by non-neoplastic melanocytes of epidermal origin was first described by Azzopardi and Eusebi in 1977. We herein report two cases on the exceptional clinical and pathological features of this phenomenon in a 66-year-old and a 51-year-old patients. The pathogenesis is not fully understood, but a disrupted basement membrane and the role of tumoral growth factors are considered essential.

## 1. Introduction

Melanocyte colonization and pigmentation of breast carcinoma was first described by Azzopardi and Eusebi in 1977 on a series comprising 14 cases [1]. This is a rare condition, and the disruption of the epidermal-dermal interface has been hypothesized as the key factor for melanocyte migration into the superficial dermis, including superficial lymphatics. Similarly, pigmentation associated to breast carcinoma is a rare phenomenon, generally more marked when the overlying epidermis is moderately to heavily pigmented as in the areolar region. The clinical significance of these findings in breast carcinoma has yet to be clarified, and specific relationships with classical pathological parameters have not been revealed.

We herein report two examples of breast carcinomas associated to pathological melanocyte migration and melanosis; then, we reviewed the literature regarding this particular topic.

## 2. Case Report and Review of Literature

The first case regards a 66-year-old woman, in good clinical conditions, that in January 2018 presented at our institution with an irregular, pigmented nodular lesion of left breast skin and parenchyma measuring 2 cm, with nipple retraction, skin thickening and hyperpigmentation, without cutaneous ulceration. She did not present lymphadenopathies. X-ray imaging revealed a hardening with irregular margins and subsequent ultrasound examination showed a 25 mm dishomogeneous hypoechoic area, corresponding at MRI to a nodule of 24 × 14 × 16 mm with retraction of nipple-areola complex. In March 2018, the patient underwent an osseous scintigraphy, that was negative, and a total body computerized tomography that confirmed the presence of a 20 mm left retroareolar nodule with contrast enhancement, in absence of repetitive lesions.

The histological examination revealed two neoplastic foci, one of 2.1 cm and the other measuring 1.6cm. The first showed a solid, glandular and papillary growth and high-grade nuclear atypia, as a G3 ductal invasive carcinoma with the following immunoprofile: ER (clone SP1 Ventana Medical Systems, Inc., Tucson, AZ, USA; 98%); PgR (clone 1E2 Ventana Medical Systems, Inc., Tucson, AZ, USA; 60%); HER2 (clone 4B5 Ventana Medical Systems, Inc., Tucson, AZ, USA; score 0 according ASCO/CAP 2018), Ki67 (clone 30-9 Ventana Medical Systems, Inc., Tucson, AZ, USA; 75%).

**Figure 1:**
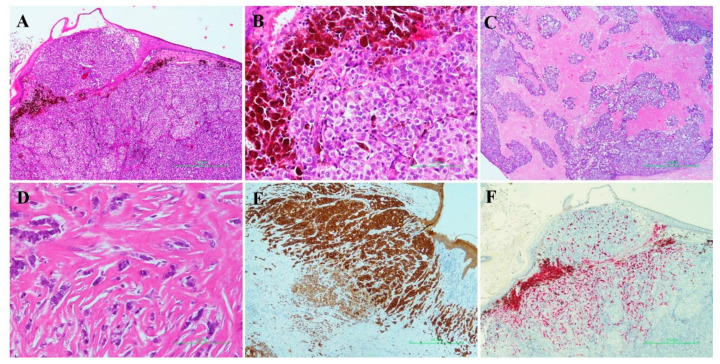
**Histological and immunohistochemical features of case 1**. (**A**) Low power view (2×) demonstrating a neoplastic proliferation growing in irregular nests and sheets beneath a thinned epithelial layer. (**B**) The neoplasm showed a nevoid appearance with abundant brown melanin pigment surrounding neoplastic cells with epithelioid morphology. (**C**) The deeper portion of the neoplasm resembled an invasive breast carcinoma with abundant tumor necrosis and no pigmentation. (**D**) Some tumor areas showed a lobular-like tumor growth constituted of single files of tumor cells loosely dispersed throughout fibrous matrix. (**E**) By immunohistochemistry, neoplastic cells showed diffuse immunoreactivity for cytokeratin AE1-AE3. (**F**) By contrast, HMB 45 staining was negative in neoplastic cells and highlighted only the colonizing melanocytes.

The other lesion was composed of epithelial neoplastic cells, sometimes with nevoid appearance, growing in irregular nests or in thin sheets, infiltrating the epidermis with a pagetoid habitus (Figure 1). Overlying epidermis was thinned. Acantholysis and tumoral necrosis were present. Massive vascular tumor embolization was seen. Abundant melanin pigment was observed in elongated and dendritic melanocytes colonizing the underlying carcinoma cells. By immunohistochemistry, neoplastic cells showed immunoreactivity for CKs (anti-Cytokeratin Pan, Clone: AE1/AE3 clone SP1 Ventana Medical Systems, Inc., Tucson, AZ, USA), GATA3 (clone L50-823 Ventana Medical Systems, Inc., Tucson, AZ, USA), ER (clone SP1 Ventana Medical Systems, Inc., Tucson, AZ, USA; 95%); PgR (clone 1E2 Ventana Medical Systems, Inc., Tucson, AZ, USA; 40%); HER2 (clone 4B5 Ventana Medical Systems, Inc., Tucson, AZ, USA; score 2 according ASCO/CAP 2018), Ki67 (clone 30-9 Ventana Medical Systems, Inc., Tucson, AZ, USA; 10%) and were negative for Melan-A, S100 (clone 4C4.9 Ventana Medical Systems, Inc., Tucson, AZ, USA) and HMB45 (Anti-Human Melanosome Clone HMB45 Ventana Medical Systems, Inc., Tucson, AZ, USA). The last two antibodies were positive only in the colonizing melanocytes. SISH analysis revealed no amplification status.

The final diagnosis was therefore of breast bifocal ductal carcinoma with melanocyte colonization and superficial pigmentation.

The second case regards a 51-year-old woman that was diagnosed with a breast high grade invasive ductal carcinoma in May 2011 and was treated with a neoadjuvant therapy (hormonal treatment and chemotherapy). This treatment induced partial regression of the tumor and the patient underwent a mastectomy with axillary dissection in October 2011. Clinically, the overlying breast skin was dyschromic with multiple heavily pigmented areas, mimicking malignant melanoma.

**Figure 2:**
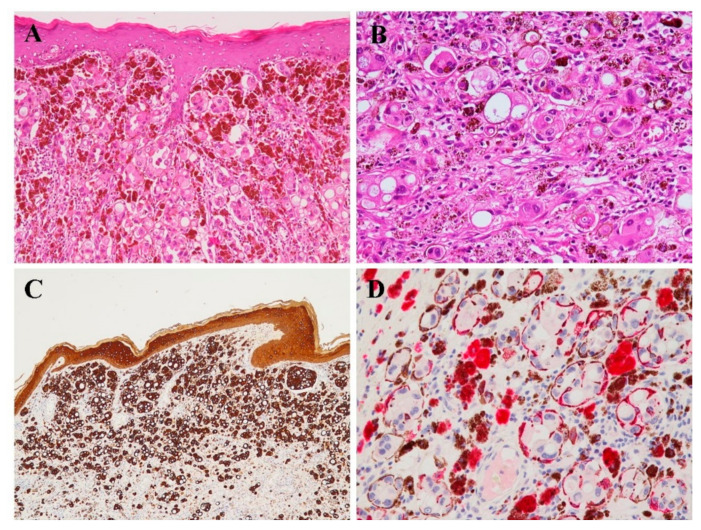
**Histological and immunohistochemical features of case 2.** (**A**) Low power view (4×) demonstrating a solid neoplastic proliferation with abundant pigment deposits, infiltrating the epidermis. (**B**) High power view (20×) demonstrating the epithelioid appearance of tumor cells showing abundant eosinophilic cytoplasm’s, intermixed with melanin with melanin pigment deposits. (**C**) The neoplasm showed a nevoid appearance with abundant melanin surrounding neoplastic cells with epithelioid morphology. (**D**) By immunohistochemistry, neoplastic cells showed diffuse immunoreactivity for cytokeratin AE1-AE3. HMB 45 staining highlighted only the surrounding melanocytes, while negative staining was observed in tumor cells.

Examination of the mastectomy specimen showed a macroscopic residual carcinoma with reactive iatrogenic pleomorphism, widespread fibrosis and ulceration of nipple-areola complex. Interestingly, there were multiple heavily pigmented non-elevated areas in the skin: in several points, in fact, elongated and dendritic melanocytes carrying abundant melanin pigment colonized the underlying neoplastic epithelial cells that had infiltrated the superficial dermis and penetrated the dermo-epidermal junction (Figure 2). In this way, neoplastic epithelial cells focally showed cytoplasmic pigmentation. Immunohistochemical analyses confirmed the morphological findings, with results similar to those described abo, regarding melanocytic markers. The biological characterization revealed the following profile: ER (clone SP1 Ventana Medical Systems, Inc., Tucson, AZ, USA; 90%); PgR (clone 1E2 Ventana Medical Systems, Inc., Tucson, AZ, USA; 90%); HER2 (clone 4B5 Ventana Medical Systems, Inc., Tucson, AZ, USA; score 0 according ASCO/CAP 2018), Ki67 (clone 30-9 Ventana Medical Systems, Inc., Tucson, AZ, USA; 5%).

Taking into consideration this unusual pigmentation phenomenon, a careful review of the literature on this topic has been performed. In detail, four electronic databases (PubMed, Scopus, Wed of Science and Google Scholar) were searched from January 1980 to March 2021. The following word combination was used: breast AND (cancer OR carcinoma) AND melanocyte AND pigmentation. In this way, 21 cases of breast carcinoma with extensive pigmentation, were retrieved from the literature (Table 1).

## 3. Discussion

In this paper, we showed how melanocytes not only can survive in the unusual environment of breast carcinoma, but also produce melanin and transfer the pigment to neoplastic cells. Colonization of non-melanocytic lesions by dendritic melanocytes is a curious phenomenon and it has been described in various epithelial tumors like oral mucosa, larynx, salivary gland, prostate gland and rectal mucosa [1,2,3,4,5,6,7,8,9,10,11,12,13,14,15,16,17,18,19,20,21,22,23]. Melanophages are a consequence of this phenomenon, resulting from the ingestion of the pigment released from both colonizing melanocytes and from cancer cells. The significance of this finding in breast carcinoma has yet to be fully clarified, but different hypotheses can be supposed and discussed below.

Since no melanocytes are normally present in mammary ductal and acinar cells, the concepts of melanocyte implant, ectopia or metaplasia are pure speculations.

Melanocytes are thought to be a stable population, showing ability to migrate and/or proliferate except in close proximity to the epidermal-dermal junction or in an environment of keratinocytes, especially of basal type. The disruption of the epidermal-dermal interface is considered a key factor for melanocyte migration into the superficial dermis, including superficial lymphatics, so that melanocytes can be passively drained to regional lymph nodes by metastasizing tumor cells.

Since transforming growth factors and epidermal growth factor receptors are involved in the proliferation of normal and neoplastic human cells, in vitro experiments have been performed to study the role of growth factors in melanocyte colonization of neoplastic cells. Activated melanocyte growth factors stimulate the proliferation of normal human melanocytes, and a possible role in this phenomenon is played by a transforming growth factor released by breast cancer cells [24,25].

There is growing evidence that also dermis can be a reservoir of human multipotent dermal stem cells (DSCs) capable of self-renewing and differentiating into a wide variety of different cell types including mesenchymal and neuronal lineages and melanocytes [26]. Other melanocyte stem cells (melanoblasts) rely in the bulge area of the hair follicles. Moreover, the observation that epidermal melanocytes molecularly differ from dermal melanocytes seems to support the hypothesis about a double origin of skin melanocytes [27], which from neural crest cells colonize the skin via a dorsolateral migratory pathway or from ventrally migrating precursors forming the myelin around the cutaneous nerves [28]. These last cells are retained in a stem cell-like state until the signal sent by the end of the cutaneous nerve promotes them to differentiate into melanocytes.

Most patients in our review were treated with surgery ± adjuvant chemotherapy ± radiotherapy ± hormonal treatment for primary breast carcinoma and all of them showed tumor recurrence with melanocyte colonization several months after the therapy was discontinued (Table 1). In one of our cases, hyperpigmentation and histologically confirmed melanocyte colonization have been observed only after neoadjuvant therapy. Therefore, we can speculate a role of chemo/radiotherapy in the genesis of melanocyte colonization of cutaneous localizations of breast carcinoma: in fact, cutaneous hyperpigmentation has been associated with many chemotherapeutic agents and also with radiation therapy [29,30]. Therefore, the disruption of the dermo-epidermal junction could be the underlying mechanism of therapy-related melanocyte colonization of breast carcinoma cutaneous recurrences.

From a pathological point of view, there is no correlation between the incidence of colonization and pathological features, such as tumor type or differentiation degree.

Due to the rarity of this migration phenomenon in the breast carcinoma, its clinicopathological aspects are largely unknown, leading to diagnostic difficulty. Possible differential diagnoses included primary or metastatic melanoma, in particular dedifferentiated forms with aberrant CK expression, collision tumors, melanotic carcinoma and carcinoma with extensive melanosis.

Primary malignant melanoma of the breast parenchyma or the overlying skin is the first to be ruled out during diagnosis [31,32]. Moreover, mammary gland can be the land of metastases in patients affected with malignant melanoma, that can be considered the most frequent type of neoplasia metastasizing to the breast tissue [33]. Melanoma cells have a variable appearance ranging from epithelioid to spindled cells and include different cytoplasmatic morphologies and often marked pleomorphism and nuclear atypia. Due to the extreme plasticity of melanomatous cells, pathological diagnosis of melanoma, in the skin as elsewhere, is challenging so much so that melanoma can simulate a primary breast carcinoma both clinically and morphologically, especially the “triple negative,” poorly differentiated form. A negative result for cytokeratin staining, associated to the positivity for melanocytic markers generally should induce the suspicion for melanoma. However, we have to remember that cytokeratin reactivity has been reported also in melanoma, in particular in dedifferentiated forms with aberrant cytokeratin expression, confirming the phenotypic plasticity of malignant melanoma [34]. Moreover, antibodies with a classically positive profile in melanoma, including S100, Melan-A, Tyrosinase, HMB-45, SOX10, and MITF, may have a variable level of sensitivity or even a negative staining. Finally, an elevated Ki-67 or Mib-1 percentage of staining can be observed in malignant melanoma as well as in high grade breast carcinoma, not aiding in differential diagnosis. There are only isolated case reports of melanoma occurring in the primary breast carcinoma as an example of cancer-to-cancer metastasis. Some Authors also reported synchronous axillary nodal involvement by melanoma and carcinoma components [35].

Some authors have reported rare cases of metaplastic carcinoma with melanocytic differentiation, characterized by a melanocytic tumoral area lacking keratin staining but positive for S100 protein and HMB-45, in the midst of CK-positive metaplastic carcinomatous cells. Histologically, this neoplasm revealed multidirectional differentiation, consisting primarily of neoplastic epithelial and melanocytic cell types, sometimes also with focal squamous or glandular and osseous metaplasia. Non-pigmented ductal carcinoma cells showed the following profile: CK+ S100- HMB-45-; while the pigmented cells were CK- S100+ HMB-45+. Based on the morphologic, immunohistochemical, and ultrastructural findings, such tumors fall within the spectrum of metaplastic carcinomas of the breast, in which carcinoma and melanoma components had arisen from the same clone with subsequent aberrant melanocytic differentiation due to the occurrence of multiple genetic alterations [36,37].

Cancer melanosis is a rare phenomenon in breast gland. The English literature reported only 21 cases of breast carcinoma with extensive pigmentation, in the absence of primary or metastatic melanoma (Table 1) [38]. Immunohistochemistry can help in the identification of melanin pigment, that results black stained by Fontana-Masson, but unstained with PAS and Prussian blue.

Our cases were histologically composed by dendritic melanocytes carrying abundant melanin pigment colonizing the surrounding neoplastic epithelial cells that had infiltrated the superficial dermis and penetrated the dermo-epidermal junction. Interestingly, similarly to the report of Mele M. et al. [11], one of the two studied cases has shown how melanocyte colonization of the neoplastic cells can persist even after neoadjuvant therapy. The histopathological diagnosis of melanocyte colonization of a breast carcinoma is complex. The application of a complete panel of antibodies, the knowledge of clinical history and the adequate clinicopathologic correlations are needed to render the correct interpretation both on surgical and bioptical samples and appropriate to define the clinical management.

## 4. Conclusions

The presence of melanocytes colonization and melanin pigmentation is described in various epithelial tumors like carcinomas of oral mucosa, larynx, salivary gland, prostate gland, and the rectal mucosa. On the other hand, the breast gland is an unusual ‘host’ for this phenomenon, maybe due to the improvement in early diagnosis of breast cancer, with only rare cases detected in T4 stage (characterized by epidermal infiltration, disruption of basal membrane and cutaneous ulceration). The exact pathogenesis of melanocyte migration is not fully understood (breached basement membrane, epidermal growth factors, tumoral growth factors). Clinical and histological examination, but also additional immunohistochemical staining are essential to differentiate breast cancer with melanocyte colonization and pigmentation from other pathological entities that can affect breast gland (malignant melanoma, melanotic breast carcinoma, breast cancer melanosis). Further studies are required to exactly understand the molecular basis of this phenomenon and the possible association to clinical and pathological data.

## Figures and Tables

**Table 1:** Literature regarding melanocyte colonization of breast carcinomas.

Author	Year	Number of Cases	Age (Years)/Sex	Breast Carcinoma Histology (± Immunohistochemistry)	Melanocyte Colonization Histology	Pigmentation in Primary Carcinoma	Pigmentation in Recurrence/Cutaneous Metastasis	Therapy	Reference
Azzopardi and Eusebi	1977	14	ND	13 invasive ductal carcinomas, 1 invasive lobular carcinoma	Involvement of dermo-epidermal junction, presence of pigment in the cytoplasm of breast carcinoma cells, proliferation of dendritic melanocytes among carcinoma cells	ND	ND	ND	[1]
Marco et al.	1988	1	45/F	Invasive ductal carcinoma	Involvement of dermo-epidermal junction, proliferation of dendritic melanocytes and numerous melanophages among neoplastic cells of mammary carcinoma	NO	YES	Surgery + chemotherapy and radiotherapy	[2]
Poiares-Baptista and Abreu de Vasconcelos	1988	1	70/F	Invasive carcinoma NOS	Involvement of dermo-epidermal junction, melanin within intraepidermal Paget cells, melanophages in papillary dermis	NO	YES	Radiotherapy	[3]
Pierard et al.	1990	1	ND	Invasive carcinoma NOS	Involvement of dermo-epidermal junction, proliferation of dendritic melanocytes among neoplastic cells of mammary carcinoma	ND	ND	ND	[4]
Konomi et al.	1992	1	65/F	Invasive papillo-tubular carcinoma, ER-	Involvement of derm-oepidermal junction, proliferation of dendritic melanocytes among neoplastic cells of mammary carcinoma	NO	YES	Surgery + chemotherapy and radiotherapy	[5]
Kutzner et al.	1992	1	53/F	Invasive carcinoma NOS	Involvement of derm-oepidermal junction, proliferation of dendritic melanocytes among neoplastic cells of mammary carcinoma	ND	ND	ND	[6]
Shamal-Lubovitz et al.	1994	1	40/F	Invasive ductal carcinoma	Involvement of dermo-epidermal junction, melanin within neoplastic cells of mammary carcinoma	NO	YES	Surgery + chemotherapy and radiotherapy	[7]
Bourlond	1994	1	79/F	Moderately differentiated invasive canalicular adenocarcinoma	Involvement of dermo-epidermal junction, proliferation of dendritic melanocytes among neoplastic cells of mammary carcinoma, melanophages in the papillary dermis	NO	YES	Surgery + chemotherapy and tamoxifen	[8]
Requena et al.	1996	2	76/F-52/F	1 invasive ductal carcinoma, 1 invasive carcinoma NOS	Involvement of dermo-epidermal junction, proliferation of dendritic melanocytes among neoplastic cells of mammary carcinoma	NO	YES	Surgery	[9]
Requena et al.	2002	6	middle age 63.8/F	Invasive carcinoma NOS	Involvement of dermo-epidermal junction, proliferation of dendritic melanocytes among neoplastic cells of mammary carcinoma, melanin within neoplastic cells	NO	YES	Only surgery in 5 patients; surgery + radiotherapy in 1 patient	[10]
Mele et al.	2012	1	74/F	Invasive carcinoma, ER+	Involvement of dermo-epidermal junction, proliferation of dendritic melanocytes among neoplastic cells of mammary carcinoma	NO	YES	Surgery + letrozole	[11]
Ubillos et al.	2016	1	67/F	Invasive ductal carcinoma	Involvement of dermo-epidermal junction, presence of pigment in the cytoplasm of breast carcinoma cells	NO	YES	Surgery + chemotherapy and radiotherapy	[12]
Koga et al.	2016	1	60/F	Inflammatory breast carcinoma	Involvement of dermo-epidermal junction, proliferation of dendritic melanocytes and melanophages among neoplastic cells of mammary carcinoma	NO	YES	Chemotherapy + hormonal treatment	[13]
Gaitan-Gaona et al.	2016	1	66/F	Invasive ductal carcinoma, ER+, PR+	Involvement of dermo-epidermal junction, presence of pigment in the cytoplasm of breast carcinoma cells, proliferation of dendritic melanocytes among carcinoma cells	YES	The patient is currently free of disease	None (a pigmented cutaneous metastasis was the first manifestation of breast carcinoma)	[14]
Ishihara-Yusa et al.	2018	1	48/F	Invasive ductal carcinoma	Involvement of dermo-epidermal junction, proliferation of dendritic melanocytes among neoplastic cells of mammary carcinoma	NO	YES	Surgery + chemotherapy	[15]
Yalici-Armagan et al.	2019	1	61/F	Invasive ductal carcinoma	ND	NO	YES	Surgery + chemotherapy and hormonal treatment	[16]

## Data Availability

The data presented in this study are available on request from the corresponding author.

## References

[1] Azzopardi J.G., Eusebi V. (1977). Melanocyte colonization and pigmentation of breast carcinoma. Histopathology.

[2] Marco V., Autonell J., Cirera L., Gay M. (1988). Breast cancer melanosis in a postmastectomy scar. Cancer.

[3] Poiares-Baptista A., Abreu de Vasconcelos A. (1988). Cutaneous Pigmented Metastasis from Breast Carcinoma Simulating Malignant Melanoma. Int. J. Dermatol..

[4] Pierard G.E., Pierard-Franchimont C., Arrese Estrada J., Ben Mosbah T. (1990). Tumeurs epitheliales a contingent melanocytaire. Annales de Dermatologie et de Vénéréologie.

[5] Konomi K., Imayama S., Nagae S., Terasaka R., Chijiiwa K., Yashima Y. (1992). Melanocyte chemotactic factor produced by skin metastases of a breast carcinoma. J. Surg. Oncol..

[6] Kutzner H., Hugel H., Embacher G. (1992). Pigmentierte Morbus Paget und pigmentierte Mammakarzinommetastase. Klinische und histologische Nachahmer maligner Melanome der Brust. Hautarzt.

[7] Shamai-Lubovitz O., Rothem A., Ben-David E., Sandbank M., Hauben D. (1994). Cutaneous metastatic carcinoma of the breast mimicking malignant melanoma, clinically and histologically. J. Am. Acad. Dermatol..

[8] Bourlond A. (1994). Pigmented Epidermotropic Metastasis of a Breast Carcinoma. Dermatology.

[9] Requena L., Yus E.S., Núñez C., White Jr C.R., Sangueza O.P. (1996). Epidermotropically metastatic breast carcinomas. Rare histopathologic variants mimicking melanoma and Paget’s disease. Am. J. Dermatopathol..

[10] Requena L., Sangueza M., Sangueza O.P., Kutzner H. (2002). Pigmented Mammary Paget Desease and Pigmented Epidermotropic Metasta-ses from Breast Carcinoma. Am. J. Dermatopathol..

[11] Mele M., Laurberg T., Engberg Damsgaard T., Funder J., Jensen V. (2012). Melanocyte colonization and pigmentation of breast carci-noma: Pathological and clinical aspects. Case Rep. Pathol..

[12] Ubillos N., Vola M., Mazzei M.E., Magliano J. (2016). Pigmented Cutaneous Meastasis of Breast Carcinoma Mimicking a Melanoma. Actas Dermosifiliogr..

[13] Koga K., Nakaura J., Imafuku S., Nabeshima K. (2017). Pigmented epidermotropic metastasis from breast carcinoma. J. Dermatol..

[14] Gaitan-Gaona F., Said M.C., Valdes-Rodriguez R. (2016). Cutaneos Metastatic Pigmented Breast Carcinoma. Dermatol. Online J..

[15] Ishihara-Yusa S., Fujimura T., Lyu C., Sugawara M., Sakamoto K., Aiba S. (2018). Breast Cancer Metastasis in the Skin with Hyperkeratotic Pigmentation Caused by Melanocyte Colonization. Case Rep. Oncol..

[16] Yalici-Armagan B., Demircan C., Elcin G. (2019). Melanoma-like pigmented cutaneous metastasis of breast carcinoma. Int. Wound J..

[17] Ruffolo E.F., Koerner F.C., Maluf H.M. (1997). Metaplastic carcinoma of the breast with melanocytic differentiation. Mod. Pathol..

[18] Varga Z., A Kubik-Huch R., Spycher M., Pok J., Caduff R. (1999). Melanosis arising in a lumpectomy scar, mimicking invasive carcinoma. Histopathology.

[19] Bartal A.H., Pinsky C.M. (1985). Malignant melanoma appearing in a post-mastectomy lymphedematous arm: A novel association of double primary tumors. J. Surg. Oncol..

[20] Rosen P.P. (1997). Anatomy. Rosen’s Breast Pathology.

[21] Rosen P.P. (1997). Inflammatory and reactive tumors. Rosen’s Breast Pathology.

[22] Rosen P.P. (1997). Cutaneous neo-plasms. Rosen’s Breast Pathology.

[23] Davies J.D. (1974). Pigmented periductal cells (ochrocytes) in mammary dysplasias: Their nature and significance. J. Pathol..

[24] Wilkins L., Gilchrest B.A., Szabo G., Weinstein R., Maciag T. (1985). The stimulation of normal human melanocyte proliferation in vitro by mela-nocyte growth factor from bovine brain. J. Cell. Physiol..

[25] Saitoh K., Saga K., Okazaki M., Maeda K. (1998). Pigmented primary carcinoma of the breast: A clinical mimic of malignant mela-noma. Br. J. Dermatol..

[26] Zabierowski S.E., Fukunaga-Kalabis M., Li L., Herlyn M. (2011). Dermis-derived stem cells: A source of epidermal melanocytes and melanoma?. Pigment. Cell Melanoma Res..

[27] Aoki H., Yamada Y., Hara A., Kunisada T. (2009). Two distinct types of mouse melanocyte: Differential signaling requirement for the maintenance of non-cutaneous and dermal versus epidermal melanocytes. Development.

[28] Sommer L. (2011). Generation of melanocytes from neural crest cells. Pigment. Cell Melanoma Res..

[29] Koppel R.A., Boh E.E. (2001). Cutaneous reactions to chemotherapeutic agents. Am. J. Med. Sci..

[30] Mitera G., Chan G., Mah K., Law R., DeAngelis C., Dent R., Chow E. (2010). A Rare Adverse Skin Reaction after 8 Gy of Radiation Therapy to the Thoracic Spine: Case Report and Review of the Literature. Curr. Oncol..

[31] Kim S.K., Kim Y.W., Youn H.J., Park H.S., Jung S.H. (2012). Primary cutaneous malignant melanoma of the breast. J. Korean Surg. Soc..

[32] He Y., Mou J., Luo D., Gao B., Wen Y. (2014). Primary malignant melanoma of the breast: A case report and review of the literature. Oncol. Lett..

[33] Bacchi C.E., Wludarski S.C., Ambaye A.B., Lamovec J., Salviato T., Falconieri G. (2013). Metastatic Melanoma Presenting as an Isolated Breast Tumor: A Study of 20 Cases With Emphasis on Several Primary Mimickers. Arch. Pathol. Lab. Med..

[34] Agaimy A., Specht K., Stoehr R., Lorey T., Märkl B., Niedobitek G., Straub M., Hager T., Reis A.C., Schilling B. (2016). Metastatic Malignant Melanoma With Complete Loss of Differentiation Markers (Undifferentiat-ed/Dedifferentiated Melanoma): Analysis of 14 Patients Emphasizing Phenotypic Plasticity and the Value of Molecular Testing as Surrogate Diagnostic Marker. Am. J. Surg. Pathol..

[35] Carswell K.A., Behranwala K.A., Nerurkar A., Gui G.P. (2006). Breast carcinoma and malignant melanoma metastasis within a sin-gle axillary lymph node. Int. Semin. Surg. Oncol..

[36] Sauder C.A., E Koziel J., Choi M., Fox M.J., Grimes B.R., Badve S., Blosser R.J., Radovich M., Lam C.C., Vaughan M.B. (2014). Phenotypic plasticity in normal breast derived epithelial cells. BMC Cell Biol..

[37] Nobukawa B., Fujii H., Hirai S., Kumasaka T., Shimizu H., Matsumoto T., Suda K., Futagawa S. (1999). Breast carcinoma diverging to aberrant melanocytic differentiation: A case report with histopathologic and loss of heterozygosity analyses. Am. J. Surg. Pathol..

[38] Zhang X., Liang Y., Wang H.-Y. (2014). Invasive ductal carcinoma of the breast associated with extensive melanin melanosis: A case report and review of the literature. Int. J. Clin. Exp. Pathol..

